# Uncovering shared genetic features between inflammatory bowel disease and systemic lupus erythematosus

**DOI:** 10.21203/rs.3.rs-5804830/v1

**Published:** 2025-03-28

**Authors:** Vikram Shaw, Jinyoung Byun, Catherine Zhu, Rowland Pettit, Jeffrey Cohen, Younghun Han, Christopher Amos

**Affiliations:** Baylor College of Medicine; University of New Mexico; Baylor College of Medicine; Harvard Medical School; Yale School of Medicine; University of New Mexico; University of New Mexico

**Keywords:** inflammatory bowel disease, autoimmune disease, systemic lupus erythematosus

## Abstract

**Background:**

Inflammatory bowel disease (IBD) is an autoimmune disease (AD) characterized by chronic, relapsing intestinal inflammation. Systemic lupus erythematosus (SLE) is a complex autoimmune disease with multisystem involvement and overactivation of both innate and adaptive immunity. The extra intestinal manifestations (EIMs) that commonly occur in IBD include many of the organ sites that are affected by SLE. ADs are often comorbid with one another and may have shared underlying genetic features and architectures contributing to their pathogenesis and disease course.

**Methods:**

We performed both epidemiological and post-genome wide association study (GWAS) analyses to investigate the shared genetic features between IBD and systemic lupus erythematosus (SLE). Specifically, we performed epidemiological association analysis in the All of Us Research Program (AoURP) and genome-wide/local genetic correlation analysis and cell-type specific SNP heritability enrichment analysis using previously published summary level data.

**Results:**

A significant epidemiologic association exists between IBD and SLE with an adjusted odds ratio (aOR) of 2.94 (95% CI: 2.45–3.53; *P* < 0.001) in a multivariable model accounting for confounders in the AoURP data. Genome-wide genetic correlation analysis in previously published summary level data demonstrated a significant genetic correlation between IBD, CD, and UC with SLE, and local genetic correlation analysis demonstrated several positive and significant correlations in local genomic regions harboring disease variants in genes common to both SLE and IBD etiology, including variants in *ELF1, CD226, JAZF1, WDFY4,* and *JAK2*. Cell-type SNP heritability enrichment analysis identified both overlapping and distinct functional categories contributing to SNP heritability across IBD phenotypes. Notably, IBD-related phenotypes demonstrated significant enrichment in T-lymphocyte functional groups while SLE signal appeared in distinct categories, such as B-lymphocytes (along with CD). Gene-level collapsing analysis of rare variants in the United Kingdom BioBank (UKBB) identified overlapping significant genes between SLE and IBD, CD, and UC.

**Conclusion:**

By leveraging several post-GWAS methods, the present study identifies shared genetic features between IBD and SLE, highlighting similarities and differences in the genetic features that contribute to the pathogenesis of each disease.

## INTRODUCTION

Inflammatory bowel disease (IBD) is an autoimmune disease (AD) with two major subtypes, Crohn’s disease (CD) and ulcerative colitis (UC). IBD is characterized by chronic, relapsing intestinal inflammation, with UC occurring primarily in the large intestine and rectum and CD occurring in any part of the GI tract.^1^ IBD etiology is multifactorial, with contributions from host genetics, the immune system, environmental risks, and the gut microbiome.^2^ Interestingly, many ADs are comorbid with each other.^3,4^ Autoimmune disease mechanisms, such as pathological exosomes involved in cytokine production and other cellular processes, have been explored as potential shared features between various ADs, including IBD.^5^ Mitophagy, a form of autophagy that selectively removes dysfunctional mitochondria, has been shown as a mechanism that may contribute to both IBD and systemic lupus erythematous (SLE).^6^ Additionally, interferons *(e.g.,* IFN-y) may play a role in the pathogenesis and disease course of both conditions.^7,8^ Understanding the shared features of ADs may provide valuable insights into shared pathogenic mechanisms with the potential to inform future therapeutic selection.

Additionally, IBD has characteristic extraintestinal manifestations (EIMs), such as erythema nodosum, pyoderma gangrenosum, uveitis, peripheral arthritis, and axial arthritis.^9^ Many of these EIMs may also overlap with the signs and symptoms of SLE. Erythema nodosum may occur in patients with SLE, or with lupus erythematosus profundus, a variant of SLE primarily affecting subcutaneous fat.^10,11^ Pyoderma gangrenosum has been associated with several systemic diseases, including an uncommon association with SLE.^12,13^ Uveitis is a more common overlapping EIM, with a prevalence of 0.1–4.8% in patients with SLE.^14^ A separate study found that ocular complications, not restricted to uveitis, may occur in up to one-third of patients with SLE, causing severe ocular morbidity.^15^ Finally, musculoskeletal involvement (e.g., arthritis) is another common manifestation of SLE and may be the onset symptom in 60–80% of cases, occur in up to 60% of disease flares, and affect up to 90% of patients.^16^ Given the overlap between IBD EIMs and many SLE symptoms, it is not surprising that there are reports of co-morbid IBD and primary SLE, though the association is uncommon and requires exclusion of infectious conditions, lupus-like reactions, visceral vasculitis, and drug-induced lupus.^17–19^

SLE is a complex autoimmune disease with multisystem involvement and overactivation of both innate and adaptive immunity.^20^ IBD and SLE are heritable diseases with known genetic risk variants. Genetics have a well-established contribution to IBD, with up to 12% of IBD patients having a family history of IBD and SNP-based heritability estimates of 20–25%.^21,22^ Our previous work has calculated the SNP-based heritability of IBD, CD, and UC to be 29.6% (± 2.6%), 41.8% (± 4.4%), and 24.5% (± 2.3%), respectively.^23^ In SLE, twin studies have suggested a heritability of 66%^24^ while SNP-based heritability estimates are around 30%^25^ and genome wide association studies (GWAS) have implicated variants associated with disease risk^26^. While case reports have described patients with comorbid IBD and primary SLE^18^, to our knowledge, an opportunity exists for further characterizing the epidemiologic and genetic overlap between the two conditions. Previous work has highlighted a substantial positive genome-wide genetic correlation between CD and UC with SLE^27^, and given that these two diseases may be contemporaneous in age of onset, treatments may be more likely to be relevant to both conditions. Additionally, identifying common genetic features may also allow for improved treatment selection in comorbid IBD-SLE.

To fill this gap, the present study leverages publicly available large-scale (GWAS) summary statistic data to examine the shared genetic architecture between IBD (including the major subtypes CD and UC) and SLE. Our work serves to compliment a recent study by Yuan *et al* that was published in *BMC Genomics*^28^ by confirming the genome-wide and local genetic correlations and providing additional functional analyses of the latter findings. We also perform epidemiologic and cell-type specific enrichment analyses, identifying similar and differential patterns of SNP heritability enrichment in cells of interest. Finally, we compare and contrast genes identified through rare-variant collapsing models using whole exome sequencing (WES) data from the United Kingdom BioBank (UKBB) between IBD and SLE.

## MATERIALS AND METHODS

### Study samples

#### All of Us Research Program

The National Institute of Health’s (NIH) All of Us Research Program (AoURP) is a prospective cohort study in the US with the goal of recruiting at least one million individuals, starting in May 2018 and still actively recruiting participants, who are traditionally underrepresented in biomedical research to provide a database for a diverse range of research questions. Participants provided informed written consent to following these procedures: https://allofus.nih.gov/about/protocol/all-us-consent-process. The database includes data on lifestyle, access to care, environment, family history, and wearables data, among others. We analyzed the electronic health record and survey data of 156,707 participants in the database, including 3,528 participants with IBD using the AoURP Registered Tier Dataset v7. Patients without available sex, BMI, or smoking data were excluded from the study. Individuals with IBD were identified using Systemized Nomenclature of Medicine (SNOMED) codes: 24526004 (IBD), 34000006 (CD), and 64766004 (UC). Individuals with SLE were identified using SNOMED 55464009. Data were accessed beginning September 1, 2023, and the authors did not have access to information that could identify individual participants during or after data collection.

#### GWAS datasets for IBD, CD, and UC

The IBD, CD, and UC summary statistics used in the present study have been previously published and are publicly accessible.^29^ As previously described in the original paper, patients diagnosed with IBD using endoscopic, histopathological, and radiological criteria were consented into the study by the original study investigators (Cambridge MREC; reference 03/5/012).^29^ Following quality control steps, 4,474 CD, 4,173 UC, and 592 IBD-unclassified cases along with 9,500 controls for 296,203 variants were analyzed, and the samples were genotyped on the Human Core Exome v12 chip.^29^ After performing various sample-level and variant-level quality control steps, the final cohort included ~1.1 million loci following SNP imputation from the HapMap3 reference panel.^29^ In the present study, IBD summary statistics include all IBD cases (CD, UC, and IBD-unclassified), CD patients only for the CD cohort, and UC patients only for the UC cohort. Data were accessed beginning July 1, 2023, and the authors did not have access to information that could identify individual participants during or after data collection.

#### GWAS datasets for SLE

The SLE summary statistics have been previously published and are publicly accessible.^30^ The SLE GWAS included 7,219 cases and 15,991 controls, including a new GWAS, a meta-analysis with a previously published GWAS, and a replication study.^30^ Informed written consent was obtained by the original study’s investigators.^30^ SLE summary statistics were accessed via the European Bioinformatics Institute GWAS Catalog (https://www.ebi.ac.uk/gwas/). The SLE summary statistics were processed and harmonized similarly to the IBD summary statistics following previously published methods.^31^ Data were accessed beginning July 1, 2023, and the authors did not have access to information that could identify individual participants during or after data collection.

#### United Kingdom BioBank – AstraZeneca PheWAS Portal

The AstraZeneca PheWAS Portal (AZPP) is publicly accessible (https://azphewas.com/), and the data have been previously described.^32^ Written consent for the United Kingdom Biobank (UKBB) was obtained at time of enrollment by the original investigators using the linked form: https://www.ukbiobank.ac.uk/media/t22hbo35/consent-form.pdf. Data were accessed beginning November 1, 2024, and the authors did not have access to information that could identify individual participants during or after data collection.

### Statistical analyses

#### Epidemiological associations via All of Us Research Program

The prevalence of SLE was calculated among the cases and controls using Pearson’s χ^2^ test. Adjusted odds ratios (aORs) were calculated in the multivariable analysis using logistic regression, and significance between continuous variables was calculated using the two-sided *t*-test. Data from this program are accessible at www.allofus.nih.gov, and this study was conducted on version 7 of the data utilizing the All of Us Researcher Workbench.

#### Estimation of genome-wide genetic correlation via LDSR

The summary statistics were harmonized (as described previously)^23,31^, with the final files each containing the following columns for downstream analysis: SNP ID, reference allele, effect allele, z-score, and sample size. Genome-wide genetic correlations were estimated via LDSR^31^, which utilizes linkage disequilibrium (LD) patterns to calculate a shared genetic basis between traits.^31,33^ Briefly, an LD score exists for each SNP in the genome capturing the pairwise LD between that SNP and every other SNP in the genome.^31^ The LD scores were derived from a HapMap3 reference panel of individuals with known genotype information, and LDSR was then utilized to calculate the genetic covariance and genetic correlation between each trait by regressing the product of SNP z-scores against the SNP’s calculated LD score.^31^ The slope of the regression provides an estimate of the genetic covariance, which is then converted into a genetic correlation value as described in detail previously.^31^ The intercept term of the regression is used to account for genomic inflation from cryptic relatedness or population stratification.^31,34^ The SNP-based heritability estimates were also included from the LDSR analysis (Supplementary Table S1).

#### Estimation of local genetic correlation via SUPERGNOVA

**Figure 1:**
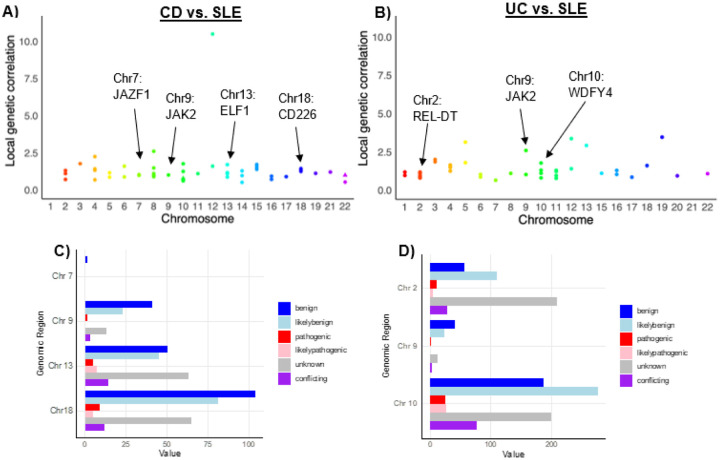
Local genetic correlation analysis for CD-SLE (**A**) and UC-SLE (**B**). Top panel: Nominally significant (*P* < 0.05) positive correlations are shown as circles while correlations achieving Bonferroni-corrected significance are indicated with triangles. Notable genomic regions harboring shared disease-specific variants are indicated with arrows and labels. Functional annotation analysis for CD-SLE (**C**) and UC-SLE (**D**). Bottom panel: Functional annotation of each highlighted genomic region using FAVOR^36^ functional annotation platform.

Local genetic correlation analysis was performed using SUPERGNOVA^35^, a statistical framework that can estimate local genetic correlations using GWAS summary statistics. While the methods are described previously in detail^35^, in brief, the program requires input summary statistics from the disease of interest, a reference panel from the 1000 Genomes Project with rare variants (minor allele frequency, MAF, < 5%) filtered out, and genome partition files specifying the local genetic regions, with the average partition size around ~ 1 million base pairs.^35^ First, the reference panel is used to generate a local LD matrix.^35^ Next, the partitioned genomic regions and a local LD matrix undergo eigen decomposition, at which point they are combined with GWAS summary statistics from the disease of interest to generate transformed z-scores.^35^ Finally, a weighted least squares regression is performed with the transformed z-scores to identify local genetic covariances.^35^ Conceptually, local genetic correlation is similar to genome-wide genetic correlation, except the focus is on SNPs within a pre-specified genomic region.^35^ The challenge, however, is that local z-scores are likely to be highly correlated due to extensive LD in local regions, and SUPERGNOVA solves that challenge through the aforementioned decorrelation of local z-scores with eigenvectors of the local LD matrix.^35^ Pairwise local genetic correlation analysis was performed for UC, CD, and SLE. Local genomic regions demonstrating a positive correlation and at least nominal (*P*< 0.05) significance were included. Bonferroni correction was also applied for both the CD-SLE and UC-SLE comparisons by multiplying the number of analyses (*n* = 2254 and *n* = 2253, respectively) by the p-value. Correlations achieving Bonferroni-corrected significance are indicated with triangles (Fig. 1A–B). Functional analysis of local genomic regions was performed in the FAVOR platform, a resource with multi-omic functional annotations for each of the nine billion single nucleotide variants in the genome.^36^

#### Cell-type specific SNP heritability enrichment (s-LDSC)

**Figure 2:**
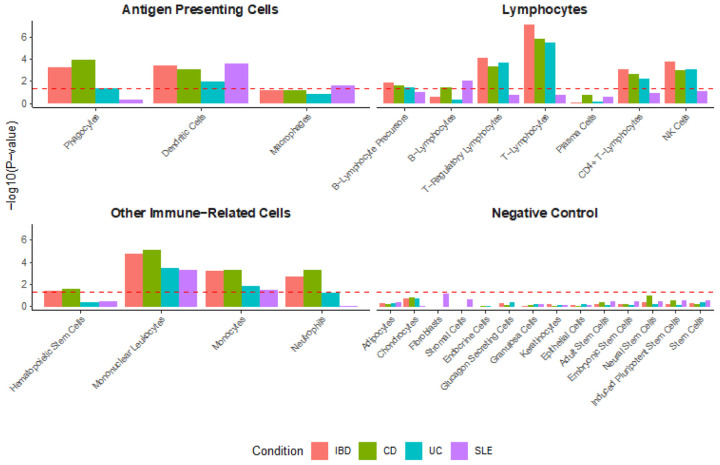
sLDSC cell-type specific partitioned heritability analysis for IBD, CD, UC, and SLE across several immune cells of interest. Red dashed line indicates *P*-value cutoff for 5% FDR-adjusted significance.

Stratified linkage disequilibrium score regression (s-LDSC) is a method for partitioning heritability and is used to test whether SNP heritability for a given disease is enriched in genes, or regions surrounding genes, with cell-type specific expression.^37^ Cell-type specific expression data processed in a previously published paper^37^ and originally found here (GTEx, http://www.gtexportal.org/) was utilized. In addition to the expression data, baseline model and standard regression weights were obtained from the s-LDSC Github (https://github.com/bulik/ldsc/wiki/Cell-type-specific-analyses).^37^ The output file contained a list of the studied cell types, along with the estimate and standard error of the first regression coefficient from the s-LDSC regression and a *P*value from a one-sided test that the coefficient is greater than zero, which is selected to test the hypothesis that the change in per-SNP heritability from a given annotation is positive.^37^ The cell-type analysis included antigen presenting cells (phagocytes, dendritic cells, and macrophages), various lymphocytes, and other immune cells (hematopoietic stem cells, mononuclear leukocytes, monocytes, and neutrophils). Fourteen putatively unrelated cell types were used as negative controls. For the cell-type analysis, an FDR-corrected *P*value cutoff (denoted by the red dashed line) was established by first generating a vector of *P*values for the analyzed cell types for each disease (*n*_rows_ = 112). Then, the “p.adjust” function with “method = fdr” was used to establish the FDR-corrected *P*values, and a cutoff line for significance was set at *P*< 0.05. The −log_10_ of this value was used to establish the dashed red cutoff line for Fig. 2.

#### Overlap of genes identified from gene-level association tests in AZPP Phewas

Methodological and statistical details on the gene-level association tests via collapsing models in the AZPP Phewas have been previously described.^32^ First, qualifying variants (QVs) are defined using model criteria, which depend on allele frequency, predicted functional consequence of the mutation, and pathogenicity scores, such as REVEL.^32,38^ Next, using the model criteria and testing 12 total models, with one serving as an empirical negative control, gene-level association tests compare the proportion of cases and controls with qualifying variants in a given gene.^32^ The full model definitions are available here: https://azphewas.com/modelDefinitions and are also available in Supplementary Table S2. P-values for the gene-level collapsing models were generated with a Fisher’s exact two-sided test.^32^ Genes with a p-value < 0.005 for both SLE and IBD, CD, or UC are presented in this analysis. The presented p-values are unadjusted and considered nominally significant as the cutoff for genome-wide significance is <1 × 10^−8^.

## RESULTS

### Epidemiological association between IBD and SLE

We first characterized the epidemiological association between IBD and SLE by performing multivariable logistic regression analysis using data from the All of Us Research Program (AoURP). A case-control study was conducted with 3,528 patients with IBD and 153,179 controls. A significant difference (*P*< 00001) in prevalence was observed between IBD patients with SLE (3.7%) compared to controls with SLE (1.4%) (Table 1). Multivariable logistic regression models controlling for age, gender, and race demonstrated an increased aOR of 2.94 in the overall cohort (95% CI: 2.45–3.53; *P =*8.6 × 10^−31^) that remained consistent across most all analyzed age groups, sexes (except “Other”), and annual household income levels (Table 2).

### Genome-wide and local genetic correlations between IBD and SLE

Next, the cross-trait genetic correlation (r_g_) was calculated between IBD, UC, and CD with SLE (Table 3). A significant positive r_g_ was seen between SLE and IBD (r_g_ = 0.19; *P =*4 × 10^−4^), CD (r_g_ = 0.13; *P =* 0.0125), and UC (r_g_ = 0.22; P =9 × 10^−4^), consistent with the recently published study by Yuan *et al*.^28^ Each of the three comparisons also achieved Bonferroni-adjusted significance.

We then performed local genetic correlation (r_g,local_) analysis to identify correlated local genomic regions that may harbor shared disease variants (Fig. 1A–B, Supplementary Table S3). These results were also consistent with those presented by Yuan *et al*^28^, though the present study discusses both nominally significant and Bonferroni-significant correlations, in addition to focusing on the positive local genetic correlations. In CD and SLE, a~1.6 million base pair genomic region on q14.11 of chromosome 13 demonstrated an r_g,local_ of 1.70 (*P =* 0.028), a region harboring a common risk variant in the *ELF1* gene (rs7329174).^39,40^ An additional positive r_g,local_ of 1.37 (*P =* 0.012) was observed on p11.31-p11.23 of chromosome 18, each harboring a different variant in the *CD226* gene. A ~ 0.67 million base pair genomic region on chromosome 7 at p15.1 demonstrated a positive r_g,local_ of 1.04 (*P =* 0.010) with each disease harboring a different risk variant in the *JAZF1* gene. A strongly significant r_g_,_local_ of 0.78 (*P =* 7.51 × 10^−9^) was also observed in CD and SLE in chromosome 10. Notably, this region contains annotated variants for *CREM,* whose CREMα isoform is a regulator of cytokine production that is implicated in SLE.^41^

In UC and SLE, a ~ 2.0 million base pair genomic region on q11.22-q11.23 of chromosome 10 demonstrated an r_g,local_ of 1.11 (*P =* 0.0430) with each disease harboring a different risk variant in the *WDFY4* gene. An additional ~ 2.0 million base pair genomic region on p16.1-p15 of chromosome 2 demonstrated an r_g,local_ of 1.08 (*P =* 0.009) with each disease harboring a different risk variant in the *REL-DT* gene. In both CD-SLE and UC-SLE analyses, a ~ 0.68 million base pair genomic region on chromosome 9 at p24.2-p24.1 with a positive r_g,local_ of 1.01 (*P =* 0.004; CD-SLE) and r_g,local_ of 1.02 (P = 0.002; UC-SLE) was observed; disease harbors > 1 risk variants in the *JAK2* gene. Of the aforementioned CD-SLE genomic regions, the chromosome 18 region appeared to contain the most annotated variants, including the greatest number of benign and pathogenic variants (Fig. 1C). In UC-SLE, the chromosome 10 region appeared to contain the most pathogenic variants (Fig. 1D).

### Cell-level SNP heritability enrichment in IBD and SLE

Next, s-LDSC^42^ was used to assess cell-level SNP heritability using GTEx data in fifteen cell types (Fig. 2). A *P*-value cutoff for 5% FDR-adjusted significance (*P* < 0.0325) was used to identify diseases with significant cell-type specific SNP heritability enrichment. No disease demonstrated significant enrichment in neural stem cells, which was used as a negative control, or plasma cells. IBD, CD, and UC demonstrated significant enrichment in T-lymphocyte functional groups, including overall T-lymphocytes, T-regulatory lymphocytes, and CD4 + T-lymphocytes. CD and SLE demonstrated significant enrichment in B-lymphocytes, and CD and IBD demonstrated significant enrichment in neutrophils. All diseases demonstrated significant enrichment in dendritic cells, and all diseases demonstrated significant enrichment in mononuclear leukocytes and monocytes. SLE demonstrated a significant enrichment in macrophages, though the enrichment for IBD and CD were close and just under the 5% FDR-adjusted significance threshold.

### Overlapping genes via gene-based analysis of UKBB WES data

Nominally-significant genes were identified via gene-based collapsing analysis of UKBB WES via the AZPP, and genes with a p-value < 0.005 for both SLE and IBD, CD, or UC are shown in Table 4. Seven, three, and two overlapping genes were identified between SLE and IBD, CD, and UC, respectively. All identified genes, except *SLC2A8* and *TNFRSF10C,* demonstrated a similar directional effect on disease risk across the studied phenotypes, and *KAZALD1, NAT10,* and *SPATA2* demonstrated consistent evidence of correlation across multiple models.

## DISCUSSION

In the present study, we leveraged publicly available GWAS summary statistic data to uncover important shared genetic features between IBD, including its two major subtypes, CD and UC, with SLE. First, we established an epidemiologic association between IBD and SLE. While studies have suggested that the diseases may share various underlying autoimmune mechanisms, such as mitophagy^6^, IL-33 signaling^43^, and interferon signaling^7,8^, to our knowledge, the epidemiological association between the two diseases has not been explored. The association is not surprising, however, given that IBD and SLE are both well-studied ADs with an underlying pathophysiology based on a self-reactive immune system. Mechanistically, autoimmunity occurs when immune tolerance is broken, allowing self-reactive lymphocytes and/or autoantibodies into the bloodstream or tissues.^44^ This process leads to inflammation, classical or pathological autoimmunity, and finally, to tissue damage.^44^

Next, we identified that IBD, CD, and UC demonstrate a positive genome-wide genetic correlation with SLE, supporting the epidemiological association with genetic evidence. Additionally, our study confirms results from Yuan *et al*. that were recently published.^28^ The evidence is also consistent with other previously published results, which estimated the CD-SLE and UC-SLE genome-wide genetic correlations at 0.15 and 0.23, respectively.^27^ The local genetic correlation analysis between CD, UC, and SLE provided additional evidence, and we identified four nominally significant local genetic correlations greater than one with a variant mapped to a common gene in CD and SLE. We identified three of these genetic correlations in UC and SLE. Both CD and SLE share a common risk variant on chromosome 13, rs7329174, which occurs in the *ELF1* gene. Variants mapped to *ELF1* have also been associated with traits including lymphocyte count^45^, neutrophil count^46^, and type II diabetes mellitus^47^, among other traits. In an SLE GWAS in an Asian cohort of 3,164 patients and 4,482 matched controls (including discovery and replication datasets), *ELF1* was found to have a positive association with an OR of 1.26 (joint *P* = 1.47 × 10^−8^).^40^ In a CD GWAS of 1,523 cases and 19,189 controls (including discovery and replication datasets) in a Japanese population, *ELF1* was found to have a positive association with an OR of 1.27 (*P* = 5.12 × 10^−9^). *ELF1* (E74-like factor 1) is a transcription factor in the ETF family and regulates a diverse range of genes that are involved in cellular processes like angiogenesis, hematopoiesis, and importantly, T-cell development and function.^40^ Additionally, *ELF1* negatively regulates Toll-interacting protein (Tollip), a negative regulator of Toll-like receptor signaling that is highly expressed in intestinal epithelial cells, further supporting the dysregulation of the immune system as a driver for disease risk in the context of an altered microbiome.^48^ This suggests that within the adaptative immune system, T-lymphocytes specifically may be at least in part responsible for the shared genetic risk of CD and SLE.

Another positive local genetic correlation between CD and SLE was observed on chromosome 7, with each disease harboring a different variant in the *JAZF1* gene, and on chromosome 9, with each disease harboring a different variant in the *JAK2* gene. SLE is associated with *JAZF1* (OR = 1.20), which is also associated with type 2 diabetes risk, prostate cancer risk, and height variation, suggesting that the gene may play a role in multiple pathways.^49^ *JAZF1* and the *JAK-STAT* pathway are associated with distal colonic CD, and studies have suggested that oral JAK inhibitors (Tofacitinib and Upadacitinib) may provide benefit in CD.^50^ The *JAK-STAT* pathway regulates a wide range of cellular processes, including immune cell development, and may contribute to AD pathogenesis as many inflammatory cytokines and interferons transduce their intracellular signals via the pathway.^51^ A positive local genetic correlation was also observed between UC and SLE on chromosome 9, and a meta-analysis found that in a wide range of studies (adult-onset, multi-age, hospital-based, and population-based), a risk variant in *JAK2* was observed in both CD and UC.^52^ Variants in JAK2 are associated with a wide range of traits, including asthma^53^, eczematoid dermatitis^53^, allergic rhinitis^53^, eosinophilic esophagitis^54^, and various lab values. Given the positive local genetic correlation between CD/UC with SLE in the region harboring the *JAK2* variant, patients with comorbid CD/UC and SLE may uniquely benefit from therapeutics targeting the *JAK-STAT* pathway, though this requires further study and investigation. Future work may also perform GWAS on patients with comorbid SLE and IBD/CD/UC, though sample size may be a limiting factor.

In UC and SLE local genetic correlation analysis, a positive local genetic correlation was observed on chromosome 10 with each disease harboring a risk variant in the *WDFY4* gene. *WDFY4* is a risk variant associated with ADs^55^, including rheumatoid arthritis^56,57^ and primary biliary cholangitis^58^. In a *WDFY4* knockout mouse model, CD8 + T-cells were reduced in the periphery and p53 activation was observed.^55^ The study suggests that a link exists between *WDFY4* and T-cells, perhaps partially explaining the observation of risk variants in the gene in both UC and SLE.

### Partitioned Heritability Analysis and Phewas Studies

We performed partitioned heritability analysis via s-LDSC to test whether SNP heritability for a given disease was enriched in genes with cell-type specific expression.^37^ This analysis extends the framework from Yuan *et al****.,*** focusing on cell-types instead of tissues.^28^ We focused on antigen presenting cells, lymphocytes, and other immune cells. Phagocytes demonstrated significant enrichment in IBD and CD while dendritic cells demonstrated significant enrichment in IBD, CD, UC, and SLE. Phagocytes, which include macrophages and neutrophils, play roles in both the innate and adaptive immune systems.^59^ Dendritic cells primarily serve as activators of the innate immune system.^60^ Interestingly, significant enrichment was seen in monocytes and natural killer (NK) cells for IBD, CD, and UC and neutrophils for IBD and CD, highlighting the role of the innate immune system in IBD specifically. This suggests that IBD (including CD and UC) has similarities and differences in innate immune cell heritability when compared to SLE.

The literature about IBD suggests that pathogenesis is driven by an abnormal T-cell response from the adaptive immune system to gut microbiota and with risk genes in innate immune system components, thereby suggesting dysfunction in both components of the immune system.^61^ Significant enrichment is seen across T-lymphocyte cell types for IBD, CD, and UC while SLE enrichment is primarily centered on B-lymphocytes. Taken together, these results highlight key potential differences in the immune cell SNP heritability in the studied diseases. Additionally, these results suggest that heritable disease risk may be focused in certain immune cells, suggesting that therapies specifically targeting these cells may be preferred.

Finally, we compared genes identified in the AZPP via gene-level collapsing analysis of rare variants in the UKBB. Rare variants often have larger effect sizes on phenotypes but may be limited by statistical power.^64^ Identification of variants with similar characteristics can improve power by allowing for gene-level collapsing analysis, as is performed in the AZPP^32^ Utilizing this analytic framework, we searched for nominally significant genes with a less stringent p-value threshold to identify those that may be shared between SLE and IBD. While the connection between some identified genes and the phenotypes was not immediately obvious, it may become clear as more proteins are characterized. Two notable overlapping genes, however, include *MMP21* and *NAT10. MMP21* is part of the matrix metallopeptidase (MMP) family, of which other MMPs have been implicated in both IBD^65^ and SLE^66^. Additionally, alterations of *NAT10* have been associated with both IBD^67^ and SLE^68^. Variation in *KAZALD1* has been associated with diseases of the eye, such as hypermetropia and myopia.^69^ Variation in *TNFRSF10C* is associated with traits including eosinophil count^70^, basophil count^45^, and leukocyte quantity^71^. *KAZALD1, NAT10,* and *SPATA2* demonstrated consistent evidence of correlation across multiple models, suggesting that they should be prioritized for future inquiry. Additionally, as more population-based whole exome sequencing data becomes available, this analysis should undergo validation to identify associations that replicate.

The present study has several limitations. First, the epidemiologic association study relied on survey data and electronic health record (EHR) data, which may be subject to biases or inaccuracies. Furthermore, too few patients were available for a more controlled (*e.g.,* controlling for additional confounding variables) association study modeling SLE. Another key limitation of this study was the primary focus on summary statistics from and the use of reference panels for European populations. While variants were identified (*e.g.,* variant in *ELF1* in Asian population^40^) that may exist across populations, this study is not completely generalizable to a larger, more diverse study population and should be repeated in other populations as more publicly accessible summary statistics and reference panels become available.

Additionally, one limitation of GWAS summary-level data is that spurious associations may arise from cryptic relatedness or population stratification. Since we rely on summary level data, we are not able to perform quality control steps related to the initial analyses that were performed. The LDSR method aims to address any systematic effects in the GWAS such as effects from cryptic relatedness by modeling an intercept term.^31,34^ Next, all of the post-GWAS downstream methods utilized in this study could benefit from additional samples and variants to increase the power. A final limitation is that it is known clinically that some IBD treatments may cause drug-induced lupus^72^, but the data available in the present study did not allow for the distinction between drug-induced lupus and primary lupus.

To conclude, the present study identifies shared genetic features between IBD and SLE. Each of these autoimmune diseases shares various genetic features that may contribute to their individual pathogenesis. We hope that this study provides a roadmap for future studies aiming to investigate the shared genetics between autoimmune diseases, with the goal of illuminating potentially shared pathways that inform future research.

## Figures and Tables

**Table 1.** Demographic and clinical characteristics of participants with IBD compared to participants without IBD for epidemiological association analysis using data from AoURP.

Characteristic	Participants, no. (%)		
	Participants with IBD (*n* = 3528)	Participants without IBD (*n* = 153,179)	P*
Current age, mean (SD)	58.3 (16.4)	57.8 (16.3)	0.11
Sex			0.41
Female	2148 (60.9)	91,815 (59.9)	
Male	1320 (37.4)	58,926 (38.5)	
Other	60 (1.7)	2438 (1.6)	
Race			< 0.00001
Asian	48 (1.4)	4845 (3.2)	
Black or African American	459 (13.0)	33,639 (22.0)	
White	2875 (81.5)	108,579 (70.9)	
Other	146 (4.1)	6116 (4.0)	
Annual household income			< 0.00001
< $50,000 / year	1484 (42.1)	72,424 (47.3)	
> $50,000 / year	2044 (57.9)	80,755 (52.7)	
BMI, mean (SD)	28.9 (7.1)	30.0 (7.8)	< 0.00001
Ever smoker			0.71
No	1982 (56.2)	86,540 (56.5)	
Yes	1546 (43.8)	66,639 (43.5)	
Systemic lupus erythematous	129 (3.7)	2091 (1.4)	< 0.00001

*SD,* standard deviation;

* *P*values calculated using Pearson’s χ^2^ test or two-sided *t*-test

**Table 2.** Multivariable logistic regression model controlling for age, gender, and race to determine the association between IBD and SLE for the overall analysis and subgroup analyses. The adjusted odds ratio for IBD is displayed in the table, modeling SLE status as the outcome.

	SLE	
	aOR (95% CI)	*P*value^+^
Overall	2.94 (2.45 – 3.53)	8.6 × 10^−31^
Age group		
30 – 39	2.83 (1.56 – 5.14)	5.9 × 10^−4^
40 – 49	3.96 (2.61 – 6.01)	1.1 × 10^−10^
50 – 59	2.73 (1.80 – 4.14)	2.5 × 10^−6^
60 – 69	2.76 (1.89 – 4.03)	1.4 × 10^−7^
^3^ 70	2.74 (1.92 – 3.91)	2.5 × 10^−8^
Sex		
Male	4.87 (3.12 – 7.59)	2.9 × 10^−12^
Female	2.69 (2.20 – 3.30)	9.8 × 10^−22^
Other	3.99 (0.90 – 17.70)	0.068
Annual household income		
< $50,000 / year	3.40 (2.68 – 4.32)	9.1 × 10^−24^
> $50,000 / year	2.45 (1.84 – 3.26)	1.1 × 10^−9^

+ *P*values calculated using logistic regression

**Table 3.** Pairwise genetic correlations (r_g_) between IBD, CD, and UC with SLE via LDSR analysis. Pb is the Bonferroni-adjusted p-value for die number of tests (*n =* 3), with adjusted significance at *P*_b_ < 0.05.

	IBD				CD				UC			
	r_g_	se	*P*	*P*_*b*_	r_g_	se	*P*	*P*_*b*_	r_g_	se	*P*	*P*_*b*_
SLE	0.19	0.05	**0.0004**	**0.0012**	0.13	0.05	**0.0125**	**0.0375**	0.22	0.07	**0.0009**	**0.0027**

**Table 4:** Overlapping genes with both SLE and IBD, CD, or UC p-value < 0.005 utilizing gene-based collapsing analysis in AZPP. Full collapsing model definitions available here: https://azphewas.com/modelDefinitions. The presented p-values are unadjusted and considered nominally significant as the cutoff for genome-wide significance is < 1 × 10^−8^.

Gene	Collapsing model (SLE)	P (SLE)	OR (SLE)	OR LCI (SLE)	OR UCI (SLE)		IBD Phenotype	P (IBD)	OR (IBD)	OR LCI (IBD)	OR UCI (IBD)
AARS2	flexdmg	0.00057	2.5882	1.5916	4.2087	rec	IBD	0.000117	2.1957	1.527	3.1573
STAC3	flexdmg	0.0029	2.963	1.5745	5.5758	raredmgmtr	IBD	0.0041	3.6195	1.701	7.7019
TMEM132C	flexnonsynmtr	0.0045	2.3143	1.381	3.8785	flexnonsynmtr	IBD	0.0031	1.5778	1.191	2.0901
SLC2A8	ptvraredmg	0.0028	3.1982	1.6419	6.2298	flexdmg	IBD	0.000348	0.445	0.272	0.7279
ZNF692	raredmgmtr	0.0037	26.5097	5.6205	125.0351	flexnonsynmtr	IBD	0.0032	2.0322	1.3307	3.1034
KAZALD1	UR	0.003	11.3729	3.4504	37.4857	ptv	IBD	0.000386	4.2907	2.1992	8.371
KAZALD1	UR	0.003	11.3729	3.4504	37.4857	ptv5pcnt	IBD	0.000958	3.7563	1.9287	7.3157
KAZALD1	UR	0.003	11.3729	3.4504	37.4857	ptvraredmg	IBD	0.0038	2.2433	1.3652	3.6861
MMP21	URmtr	0.0025	12.248	3.6996	40.5488	raredmg	IBD	0.000911	3.4736	1.8471	6.5322
NAT10	flexdmg	0.000158	3.0256	1.8318	4.9974	flexdmg	CD	0.0019	2.1897	1.3893	3.4514
NAT10	ptvraredmg	0.000207	3.2308	1.8897	5.5236	flexdmg	CD	0.0019	2.1897	1.3893	3.4514
SLC2A8	ptvraredmg	0.0028	3.1982	1.6419	6.2298	flexdmg	CD	0.000387	0.0883	0.0124	0.6279
TNFRSF10C	syn	0.0033	10.9806	3.338	36.1208	ptv5pcnt	CD	0.001	0.3112	0.1395	0.6944
NAT10	UR	0.000785	5.8594	2.5682	13.3686	flexdmg	CD	0.0019	2.1897	1.3893	3.4514
NAT10	URmtr	0.0045	6.3422	2.3071	17.4347	flexdmg	CD	0.0019	2.1897	1.3893	3.4514
AARS2	flexdmg	0.00057	2.5882	1.5916	4.2087	rec	UC	2.33E-05	2.1977	1.5831	3.051
SPATA2	ptv	0.0018	42.417	8.2171	218.9591	ptvraredmg	UC	0.0011	2.8919	1.6291	5.1336
SPATA2	ptv5pcnt	0.0018	42.417	8.2171	218.9591	ptvraredmg	UC	0.0011	2.8919	1.6291	5.1336

## Data Availability

Data from the NIH *AoURP* are publicly accessible (www.allofus.nih.gov). IBD, CD, UC, and SLE summary statistics are publicly accessible via the European Bioinformatics Institute GWAS Catalog (https://www.ebi.ac.uk/gwas/) and through the IEU Open GWAS Database (https://gwas.mrcieu.ac.uk/). The AstraZeneca PheWAS Portal (AZPP) is publicly accessible (https://azphewas.com/).
